# Non-invasive mechanical ventilation after heart surgery in children

**DOI:** 10.1186/s12890-016-0334-x

**Published:** 2016-11-29

**Authors:** Sarah Fernández Lafever, Blanca Toledo, Miguel Leiva, Maite Padrón, Marina Balseiro, Angel Carrillo, Jesús López-Herce

**Affiliations:** 1 Pediatric Intensive Care Unit, Hospital General Universitario Gregorio Marañón, Calle Dr. Castelo 47, 28009 Madrid, Spain; 2 Gregorio Marañon University Hospital Biomedical Research Foundation, Research Network on Maternal and Child Health and Development II (Red SAMID II), Madrid, Spain; 3 Department of Pediatrics, School of Medicine, Complutense University of Madrid, Madrid, Spain

**Keywords:** Non-invasive ventilation, Heart surgery, Children

## Abstract

**Background:**

The purpose of the study was to analyze the characteristics and evolution of non-invasive mechanical ventilation (NIV) in the postoperative period of heart surgery in children.

**Methods:**

Retrospective observational study including all children requiring NIV after heart surgery in a single center pediatric intensive care unit (PICU) between 2001 and 2012. Demographic characteristics, ventilation parameters and outcomes were registered, comparing the first 6 years of the study with the last 6 years.

**Results:**

935 children required invasive or non-invasive mechanical ventilation, of which 200 (21.4) received NIV. The median duration of NIV was 3 days. Mortality rate was 3.9%. The use of NIV increased from 13.2% in the first period to 29.2% in the second period (*p* <0.001). Continuous positive airway pressure (CPAP) was the most common modality of NIV (65.5%). The use of bilevel positive airway pressure mode (BIPAP) increased from 15% in the first period to 42.9% in the second period (*p* < 0.001). The nasopharyngeal tube was the most common interface (66%), but the use of nasal cannula increased from 3.3 to 41.4% in the second period (*p* < 0.001). NIV failed in 15% of patients. The mortality rate did not change, the duration of NIV decreased and the PICU length of stay increased throughout the study.

**Conclusions:**

NIV is increasingly being used in the postoperative period of heart surgery in our center with an 85% success rate and is associated with a lesser need for invasive mechanical ventilation. CPAP was the most common modality and the “nasopharyngeal tube” was the most common interface in our study although, in the latter years, the use of BIPAP and nasal cannula has increased significantly.

## Background

Outcomes in children after heart surgery depend on the severity and complexity of the underlying heart disease, preexisting conditions, duration and complications during surgery and clinical evolution in the postoperative period [1].

The use of mechanical ventilation in the postoperative period has an important impact on hemodynamics and clinical evolution [2, 3]. Several studies have found an association between mechanical ventilation in children after heart surgery and an increased risk of mortality, longer pediatric intensive care unit (PICU) length of stay (LOS) and greater costs [4, 5].

For all these reasons, an early extubation is recommended if the hemodynamic situation after surgery is acceptable [6]. Several studies have analyzed the factors that are associated with a prolonged extubation after heart surgery in children [7, 8], but there are no studies that analyze whether there is an association between NIV and the duration of invasive mechanical ventilation (IMV) and PICU LOS.

The aim of this study was to analyze the characteristics and evolution of pediatric patients treated with NIV after heart surgery.

## Methods

A retrospective review examined the medical records of all patients between 3 days and 16 years of age requiring IMV or NIV after heart surgery in a single center PICU between the 1st of January 2001 and the 31st of December 2012. The study was approved by the Gregorio Marañón University Hospital Research Ethics Committee. The physician in charge of the patient established the indication and the settings of mechanical ventilation (NIV and IMV).

The following data were collected from each patient: age, sex, indication and duration of IMV and NIV, modality of NIV, continuous positive airway pressure (CPAP) or bilevel positive airway pressure (BIPAP), interface (nasal cannula, endotracheal tube in a nasopharyngeal position (“nasopharyngeal tube”), nasal mask or full face mask), NIV failure, mortality and PICU LOS. Some authors discuss whether CPAP should be considered as non-invasive mechanical ventilation but, in this study, CPAP and BIPAP are considered non-invasive modalities of mechanical ventilation.

The indication of NIV was classified into two groups: prophylactic, immediately after extubation and non-prophylactic (patients with acute respiratory failure (ARF)). Specific scales of respiratory distress were not used. The presence of respiratory failure as well as the need for intubation were based on the physician-in-charge’s assessment of the patient.

The study period was divided into two groups (the first 6 years and the second 6 years) in order to study the evolution of respiratory support.

Diaphragmatic paralysis is defined as unilateral or bilateral dysfunction of the diaphragm. Initial suspicion was based on X-Ray findings (elevated diaphragm) or clinical findings (asymmetrical hemithorax excursion, abnormal breathing pattern using accessory respiratory muscles and paradoxical abdominal wall retraction during inspiration). Clinical suspicion was confirmed with M-mode ultrasonography and electromyography.

The program SPSS 18.0 was used for data analysis. Quantitative variables are expressed as medians and interquartile ranges (IQR) as data do not follow a normal distribution, and non-parametric tests were used for comparisons (Chi-square test for randomness with categorical outcomes, Man-Whitney test for comparing quantitative variables between two groups and Kruskal-Wallis test for comparing quantitative variables between more than two groups). A *p* value less than 0.05 was considered statistically significant.

## Results

Nine hundred and thirty five patients between 3 days and 16 years of age required mechanical support after heart surgery, with a median age of 9 months (IQR 4.0–60.0). 59.1% were males. The median duration of IMV was 1.5 days (IQR 0.5–7.0). The median LOS was 7.0 days (4.0–15.0), with a mortality rate of 3.9%. 200 patients required NIV (21.4%) and 153 patients (16.4%) received both IMV and NIV. Mean duration of NIV was 3 days (IQR 2.0–5.0).

### Characteristics of non invasive mechanical ventilation

Prophylactic NIV was the most common indication (67%), followed by ARF (33%). CPAP was the most common modality of NIV (65.5%) and the “nasopharyngeal tube” was the most common interface (66%), followed by nasal cannula (30%), full face mask (3.5%) and nasal mask (3%).

The median age of children with NIV was 6 months (IQR 3.0–24.0), the duration of IMV was 8 days (4.0–14.5), PICU LOS was 17 days (9.0–32.0) and mortality rate was 3.4% (8 patients).

### Evolution of non invasive mechanical ventilation

**Fig. 1:**
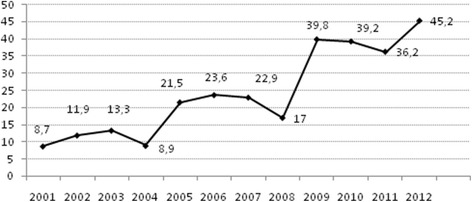
Evolution in the use of non-invasive ventilation (NIV) (percentage of patients with NIV)

The study period was divided into two groups (the first 6 years and the second 6 years) in order to study the evolution of respiratory support (Table 1). The number of patients in each study period was very similar. The use of IMV decreased significantly and NIV increased in the second period, with no variations in its indication. There were no significant differences in mortality between both periods (3.7 vs 4.0%, *p* = 0.867) but mortality tended to decrease in patients with NIV (5.8% vs 2.4%, *p* = 0.239). The use of bilevel positive airway pressure mode (BIPAP) increased from 15% in the first period to 42.9% in the second period (*p* < 0.001). The nasopharyngeal tube was the most common interface (66%), but the use of nasal cannula increased from 3.3 to 41.4% in the second period (*p* < 0.001), with a significant decrease in the use of nasal masks. The duration of NIV and the PICU LOS in patients with NIV significantly decreased in the second period. The use of NIV significantly increased throughout the study period (*p* <0.001) (Fig. 1). There was a trend toward a higher RACHS score in patients requiring NIV in the second period of the study (2.0 [2.0–3.0] vs 3.0 [2.0–3.0]) as were the number of heart transplants (8 vs 13), although these results were not statistically significant (*p* = 0.91 and *p* = 0.452, respectively).

**Table 1:** Comparison between study periods

	2001**–**2006% (*n*)	2007**–**2012	*p*
Number of patients	48.7% (455/935)	51.3% (480/935)	
Age (months)	7 (IQR 4–48)	12 (IQR 5–60)	<0.001
IMV	96.7% (440/455)	93.3% (448/480)	<0.001
NIV	13.2% (60/455)	29.2% (140/480)	<0.001
NIV only	25% (15/60)	22.9% (32/140)	0.321
NIV indication			
- Prophylactic	65% (39/60)	67.9% (95/140)	0.744
- Acute Respiratory Failure	35% (21/60)	32.1% (45/140)	
NIV type			
- CPAP	85% (51/60)	57.1% (80/140)	0.001
- BIPAP	15% (9/60)	42.9% (60/140)	
Interface			
- Nasopharingeal tuve	85% (51/60)	57.9% (81/140)	0.001
- Nasal mask	10% (6/60)	0.7% (1/140)	0.003
- Full face mask	1.6% (1/60)	3.5% (5/140)	0.417
- Nasal cannula	3.3% (2/60)	41.4% (58/140)	0.001
NIV failure	20% (12/60)	12.9% (18/140)	0.201
Mortality	3.7% (17/455)	4.0% (19/480)	0.867
	Median (IQR)	Median (IQR)	
Duration of IMV (days)	1.0 (0.5–6.0)	2.0 (0.75–7.0)	0.139
Duration of NIV (days)	3.0 (2.0–5.0)	2.0 (1.0–4.5)	0.024
PICU LOS All patients (days)	6.0 (4.0–14.0)	7.0 (4.0–15.7)	0.053
PICU LOS Patients NIV (days)	22.0 (9.0–35.0)	14.0 (8.0–23.0)	0.004

*BIPAP* bilevel positive airway pressure, *CPAP* continuous positive airway pressure, *IMV* invasive mechanical ventilation, *NIV* non-invasive ventilation, *PICU LOS* pediatric intensive care unit length of stay

### Comparison between invasive and non invasive mechanical ventilation

PICU LOS in patients receiving only IMV (5.0, IQR 3.0–10.0) was significantly lower than those with only NIV (7.0, IQR 6.0–13.0; p 0.001). PICU LOS of patients requiring both IMV and NIV (19.0, IQR 11.0–32.5) was significantly higher than those with only IMV or NIV (p 0.001).

There was a trend to a higher mortality rate in patients receiving only IMV (4.8%) than those receiving NIV (0.0%) or both (3.2%), but differences were not significant (p 0.274).

### Comparison according to age, indication and type of non-invasive mechanical ventilation

The median age of children with prophylactic NIV (18.5 months), with CPAP (8.4 months) and “nasopharyngeal tube” (6.9 months) was significantly lower than the median age of those with non-prophylactic NIV (33.1 months) (*p* = 0.002), with BIPAP (48.4 months) (*p* = 0.001) and full face mask (61.5 months) or nasal cannula (60.5 months) (*p* = 0.001).

A comparison was also made between two age groups: younger than 12 months (infants) and older than 12 months (children) (Table 2).

**Table 2:** Comparison between age groups

	<12 months (*n =* 515)	≥12 months (*n =* 420)	*P*
Periods			
- 2001–2006	54.1% (279)	41.9% (176)	<0.001
- 2007–2012	45.9% (236)	58.1% (244)	
IMV	97.6% (488)	95.2% (400)	1.0
NIV	26.7% (138)	14.8% (62)	<0.001
NIV only	5.2% (27)	4.9% (20)	0.107
NIV indication			
- Prophylactic	71.7% (99)	56.4% (35)	0.026
- Acute respiratory failure	28.2% (39)	43.5% (27)	
NIV type			
- CPAP	84.0% (116)	24.2% (15)	<0.001
- BIPAP	16.0% (22)	75.8% (47)	
Interface			
- Nasopharyngeal tube	84.0% (116)	25.8% (16)	<0.001
- Nasal mask	2.2% (3)	6.5% (4)	
- Full face mask	0%	11.3% (7)	
- Nasal cannula	13.8% (19)	64.5% (40)^a^	
NIV failure	18.8% (26)	24.2% (15)	0.247
Mortality	4.7% (24)	2.9% (12)	0.104
	Median (IQR)	Median (IQR)	
IMV days	3.0 (1.0–8.0)	1.0 (0.4–4.0)	<0.001
NIV days	3.0 (1.0–5.0)	2.0 (1.0–6.)	0.572
PICU LOS (days)	8.0 (5.0–16.7)	5.0 (3.0–12.0)	<0.001
NIV GROUP			
IMV days	8.0 (4.0–16.75)	9.0 (3.0–16.0)	0.9
PICU LOS (days)	16.0 (8.0–27.7)	15.5 (9.0–31.0)	0.9

*BIPAP* bilevel positive airway pressure, *CPAP* continuous positive airway pressure, *IMV* invasive mechanical ventilation, *NIV* non-invasive ventilation, *PICU LOS* pediatric intensive care unit length of stay

^a^The sum of the patients older than 12 months with different interfaces is 67 instead of 62 because 5 of these patients had several interfaces

The need for IMV was similar in both age groups, but infants required NIV more frequently. CPAP was the most common modality in infants and BIPAP in the older children. The “nasopharyngeal tube” was used mostly in infants and nasal cannula in older children.

Duration of IMV and PICU LOS was significantly higher in infants. There were no significant differences in the duration of NIV between both age groups.

### Non invasive mechanical ventilation failure

41 patients (20.5%) treated with NIV required intubation. 11 (5.5%) for different procedures and 30 (15%) due to NIV failure, caused by ARF in 86.7% of them and by hemodynamic instability in 13.3%.

Diaphragmatic paralysis was present in one third of the patients in which NIV failed (10 patients).

NIV failure was greater in patients that had not received IMV prior to NIV, in those intubated for ARF, in those with BIPAP and in those requiring several interfaces, nasal mask or full face mask (Table 3).

**Table 3:** NIV failure according to IMV prior to NIV, indication and type of NIV

	NIV failure (%)	*p*
IMV prior to NIV		
Yes	22/134 (16.4%)	0.046
No	19/66 (28.7%)	
Indication		
Prophylactic	22/134 (16.4%)	0.046
Respiratory failure	19/66 (28.7%)	
Modality		
CPAP	20/131 (15.2%)	0.016
BIPAP	21/69 (30.4%)	

*BIPAP* bilevel positive airway pressure, *CPAP* continuous positive airway pressure, *IMV* invasive mechanical ventilation, *NIV* non-invasive ventilation

The median duration of NIV was shorter in those patients in which NIV failed (2 days, IQR 0.88–3) than in those in which NIV was effective (3 days, IQR 2–5) (*p* = 0.002). PICU LOS was longer in patients with NIV failure (26.5 days, IQR 17.75–40.25) than in those with NIV success (14.0 days, IQR 7–25) (*p* < 0.001).

## Discussion

Mechanical ventilation modifies lung pressures and volumes affecting preload, afterload, contractility and heart rate. Positive airway pressure may reduce pulmonary vascular resistance if it achieves appropriate alveolar recruitment and it diminishes left ventricle afterload by increasing transmural pressure. On the other hand, it decreases right ventricle preload and it increases its afterload [9].

On the counterpart, IMV increases the risk of airway damage, lung injury and infection and it is associated with a longer PICU LOS [10].

An early extubation reduces the incidence of mechanical ventilation-related complications and it minimizes the undesired effects on heart function [11].

NIV decreases the risk of mechanical ventilation-related complications while maintaining the beneficial heart and lung effects of positive airway pressure, enabling an earlier extubation [12–14]. Nevertheless, very few studies analyze the utility of NIV in the postoperative period of heart surgery in adults [2, 3] or children [14–16]. Our study is the first one to analyze NIV after heart surgery in children over a long period.

Our study shows that the use of IMV has decreased as the use of NIV has increased in our unit. This has not affected the incidence of NIV failure. The use of BIPAP, which offers greater respiratory support, as well as the use of nasal cannula (which are comfortable and well tolerated) has increased throughout the study, while the use of nasal and full-face masks has been reserved for patients with a more severe ARF.

Some of the most important factors for the success of NIV are the good tolerance and acceptance of the technique on behalf of the patients as well as an increasing experience of the PICU team.

Infants require longer mechanical ventilation and their need for NIV was greater than that of older children. This fact has been described in previous studies [6, 7]. It may be due to the greater complexity of the underlying heart disease and the surgery, to their greater metabolic requirements or to their greater dependence on diaphragmatic muscles (which can become impaired after heart surgery).

The indication for NIV was prophylactic after extubation in most of our patients for having ARF or heart failure risk factors [16]. It was not possible to analyze the efficacy of NIV in reducing the risk of failure and intubation due to the characteristics of our study. Our percentage of NIV failure is consistent with what has been published by other authors [13, 16]. We are not able to answer the question of why NIV failure is greater with BIPAP due to the retrospective nature of our study. An important finding of our study is that the incidence of NIV failure remains stable even though overall use of NIV has significantly increased, and particularly in patients with respiratory distress. Thus, we think that patients that would have required intubation and mechanical ventilation in the first study period were successfully treated with NIV in the second period of the study.

This is why prospective comparative studies are required to assess whether prophylactic NIV after extubation decreases the incidence of ARF and re-intubation in children after heart surgery.

Patients requiring NIV had longer PICU LOS than those requiring only IMV, which could be misinterpreted as NIV prolonging PICU LOS. The use of NIV increased during the late study period, as did the LOS, IMV duration and the use of BIPAP. RACHS Score only takes into account surgical complexity, but not the severity of the patient’s condition before and after surgery. Even so, RACHS score and the number of heart transplants was higher for patients requiring NIV in the second period of the study (although not statistically significant). All these facts may reflect an increase in the complexity and severity of illness in the second period of the study.

Our study has several limitations: it is a retrospective review including patients with a wide variety of heart diseases. Thus, the presence of certain risk factors affecting the need for and duration of mechanical ventilation, PICU LOS and mortality were not studied, such as comorbidity or changes in postoperative care [17]. The parameters and complications of NIV were not registered either [18].

## Conclusions

NIV is increasingly being used in the postoperative period of heart surgery in our center with an 85% success rate and is associated with a lesser need for IMV. CPAP was the most common modality and the “nasopharyngeal tube” was the most common interface in our study although, in the latter years, the use of BIPAP and nasal cannula has increased significantly. Prospective multicenter studies are needed to better assess the association of NIV and the need for mechanical ventilation, PICU LOS and mortality.

## Abbreviations

ARF: Acute respiratory failure
BIPAP: Bi-level positive airway pressure
CPAP: Continuous positive airway pressure
IMV: Invasive mechanical ventilation
IQR: Interquartile range
LOS: Length of stay
NIV: Non invasive ventilation
PICU: Pediatric intensive care unit
RACHS Score: Risk adjustment for congenital heart surgery
